# Transcriptome Response of Metallicolous and a Non-Metallicolous Ecotypes of *Noccaea goesingensis* to Nickel Excess

**DOI:** 10.3390/plants9080951

**Published:** 2020-07-28

**Authors:** Agnieszka Domka, Piotr Rozpądek, Rafał Ważny, Roman Jan Jędrzejczyk, Magdalena Hubalewska-Mazgaj, Cristina Gonnelli, Jubina Benny, Federico Martinelli, Markus Puschenreiter, Katarzyna Turnau

**Affiliations:** 1 Małopolska Centre of Biotechnology, Jagiellonian University in Kraków, Gronostajowa 7a, 30-387 Kraków, Poland; piotr.rozpadek@uj.edu.pl (P.R.); rafal.wazny@uj.edu.pl (R.W.); roman.jedrzejczyk@uj.edu.pl (R.J.J.); 2 Jerzy Maj Institute of Pharmacology Polish Academy of Sciences, Smętna 12, 31-343 Kraków, Poland; mzaba1984@gmail.com; 3 Department of Biology, University of Florence, via G. La Pira 4, 50121 Florence, Italy; cristina.gonnelli@unifi.it (C.G.); federico.martinelli@unifi.it (F.M.); 4 Department of Agricultural, Food and Forest Sciences—Università degli Studi di Palermo, 90128 Palermo, Italy; jubina.benny@unipa.it; 5 Department of Forest and Soil Sciences, Institute of Soil Research, University of Natural Resources and Life Sciences Vienna, Konrad-Lorenz Straße 24, 3430 Tulln, Austria; markus.puschenreiter@boku.ac.at; 6 Institute of Environmental Sciences, Jagiellonian University in Kraków, Gronostajowa 7, 30-387 Kraków, Poland; katarzyna.turnau@uj.edu.pl

**Keywords:** *Noccaea goesingensis*, nickel (Ni), ecotypes, tolerance, transcriptome

## Abstract

Root transcriptomic profile was comparatively studied in a serpentine (TM) and a non-metallicolous (NTM) population of *Noccaea goesingensis* in order to investigate possible features of Ni hyperaccumulation. Both populations were characterised by contrasting Ni tolerance and accumulation capacity. The growth of the TM population was unaffected by metal excess, while the shoot biomass production in the NTM population was significantly lower in the presence of Ni in the culture medium. Nickel concentration was nearly six- and two-fold higher in the shoots than in the roots of the TM and NTM population, respectively. The comparison of root transcriptomes using the RNA-seq method indicated distinct responses to Ni treatment between tested ecotypes. Among differentially expressed genes, the expression of *IRT1* and *IRT2*, encoding metal transporters, was upregulated in the TM population and downregulated/unchanged in the NTM ecotype. Furthermore, differences were observed among ethylene metabolism and response related genes. In the TM population, the expression of genes including *ACS7*, *ACO5*, *ERF104* and *ERF105* was upregulated, while in the NTM population, expression of these genes remained unchanged, thus suggesting a possible regulatory role of this hormone in Ni hyperaccumulation. The present results could serve as a starting point for further studies concerning the plant mechanisms responsible for Ni tolerance and accumulation.

## 1. Introduction

Plants able to grow and reproduce on potentially toxic metal-rich soils without symptoms of toxicity are termed as “metallophytes” [1]. Tolerance to metal toxicity has evolved independently several times in members of distinct families [2]. Metallophytes have developed a range of cellular mechanisms to cope with metal toxicity as far as metal concentration in the soil remains within tolerance ranges [3,4]. This adaptation may lead to the speciation and evolution of endemic taxa, occurring solely on polluted sites and termed as obligate metallophytes [5,6]. However, most metallophytes also inhabit non-polluted environments and hence are termed pseudometallophytes or facultative metallophytes [1,7]. Most metallophytes are metal excluders that limit metal uptake and translocation into shoots (reviewed by Bothe and Słomka [8]). However, in some plant species, metals may be accumulated in shoots in concentrations beyond those present in roots (reviewed by Krämer [9]). This adaptation to metal toxicity evolved in a specific group of plants called metal hyperaccumulators. These plants can accumulate toxic metals over an element-specific threshold within their above-ground parts (e.g., for Ni above 1000 µg g^−1^) without harmful effects that prevent them from maintaining self-sustaining populations [1]. Up to now, hyperaccumulators comprise of close to 700 plant species, most of which were found to hyperaccumulate Ni, probably due to the large distribution of geogenically Ni-enriched serpentine soils worldwide [10]. *Noccaea goesingensis* (formerly *Thlaspi goesingense* (Hálácsy) F.K.Mey) was first reported as Ni hyperaccumulating plant by Reeves and Brooks [11] and since then it was studied in regard to metal hyperaccumulation and the possibilities for its utilization in phytoremediation [12,13,14,15,16]. Metal hyperaccumulation and associated hypertolerance involve complex alterations in the plant metal homeostasis, including the enhancement of root metal uptake rates and metal mobility in root symplast, and further in the process of xylem loading, metal detoxification, distribution and sequestration in shoots (reviewed by Krämer, [9]). To date, the few studies concerning *N. goesingensis* indicated an efficient sequestration of Ni in the vacuoles in shoot cells as one of the mechanisms developed to cope with Ni toxicity [17,18]. Moreover, the metal tolerance proteins (MTPs) from *N. goesingensis* were characterised [19], revealing that genes encoding MTPs are highly expressed in this plant species and thus probably involved in Ni sequestration in vacuoles. The investigations of Freeman et al. [20,21] indicated elevated levels of glutathione in *N. goesingensis* exposed to Ni, leading to enhanced plant stress tolerance. Himmelbauer et al. [22], analysing natural populations of *N. goesingensis* collected from a serpentine site near Redlschlag, found that the root system was abundant in fine roots, probably to allow an enhanced Ni uptake from the soil. Despite the progress made in the understanding of mechanisms responsible for Ni hyperaccumulation in *N. goesingensis,* the knowledge concerning the regulatory pathways that control these mechanisms, remains still unclear. The comparison of transcriptomes in metallicolous and non-metallicolous ecotypes could represent a useful tool in such a context. To date, such research has been carried out for *N. caerulescens* (J. Presl et C. Presl) in terms of Zn tolerance with the use of microarray [23] and for general metal tolerance (for Cd, Zn and Ni) in a study targeted at root transcriptome comparison of three metallicolous populations by RNA-seq [24]. Similar studies were also performed for *Arabidopsis halleri* (L.) [25].

The mechanisms utilized by plants to deal with metal toxicity are strictly regulated on various levels, predominately by phytohormones [26,27,28,29]. Ethylene was found to be a key player in plant signalling networks in response to abiotic stress factors, including toxic metals (reviewed by Khan et al. [30]). The role of ethylene in plant tolerance to metal toxicity has been frequently described in various experimental systems [28,31,32,33]. Moreover, existing studies indicate the role of ethylene in the iron starvation response, through the interaction of ethylene responsive transcription factors EIN3/EIL1 with FIT, leading to increased FIT abundance and induced expression of genes required for Fe acquisition [34], which were also frequently shown to be involved in toxic metal uptake and distribution [25,35]. The role of ethylene in the determination of metal hyperaccumulating plant phenotype has not been unambiguously investigated and described; however, existing studies suggested that it may be one of the factors involved in this process [31,36].

The aim of this work was to identify possible mechanisms involved in Ni hyperaccumulation, with a particular regard to those relying on qualitative/quantitative changes in pathways involved in metal uptake and compartmentalization, in *N. goesingensis*. We hypothesized that *N. goesingensis* occurring naturally on a Ni-rich serpentine soil will be better adapted to Ni toxicity in comparison to plants growing on a non-metalliferous soil, which may be expressed as a higher Ni accumulation capacity and also as more efficient Ni distribution (mainly into above-ground organs). This adaptation, as the effect of long-term, permanent plant exposure to Ni, is probably caused by the qualitative/quantitative changes in pathways involved in Ni uptake and compartmentalization. To determine the mechanisms responsible for Ni hyperaccumulation, we compared the Ni accumulation capacities of two *N. goesingensis* populations, one from a serpentine soil (TM) and one from a non-metalliferous soil (NTM), and we employed the RNA-seq approach to analyse the global transcriptome response in the roots of plants growing in pot cultures supplemented with Ni.

## 2. Results

### 2.1. Plant Growth Response to Nickel Supplementation and Ni Accumulation

**Figure 1:**
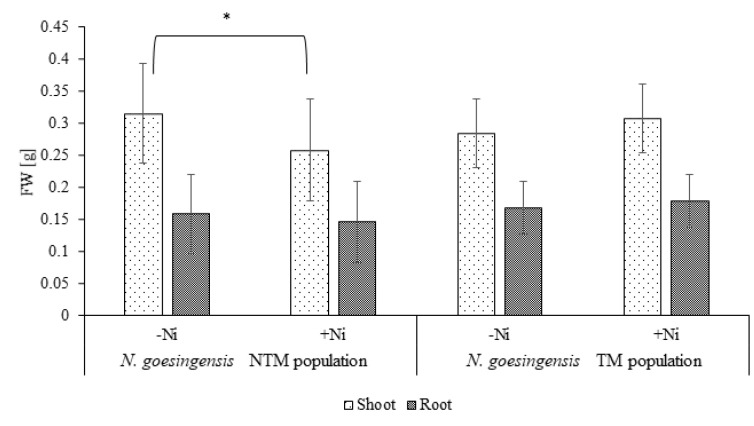
Fresh biomass of roots, shoots and whole plants of both *N. goesingensis* ecotypes in control and Ni treatment conditions. The asterisk indicates the statistical significance of the t-test at *p* ≤ 0.05, error bars represent ± SD (N = 15).

Plants from a non-metallicolous population (NTM) and from a serpentinite area (TM) were grown in control media and under Ni exposure. The fresh weight of roots of both tested populations remained unaffected by Ni in comparison to the respective untreated control. The fresh weight of shoots of the NTM population was significantly lower when grown in Ni in comparison with the untreated control, in contrast to the serpentine TM population, where there were no differences in the fresh weight of shoots in relation to control (Figure 1).

**Figure 2:**
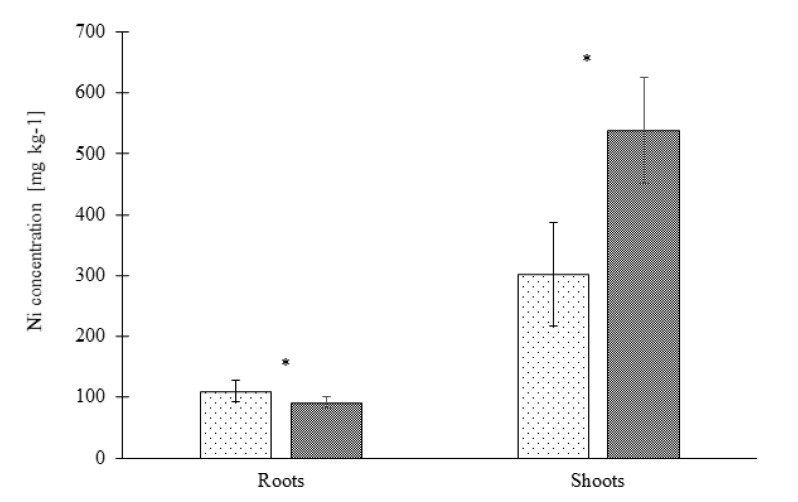
Nickel accumulation in shoots and roots of two *Noccaea goesingensis* ecotypes upon 21 days of exposure to 150 µM Ni. Error bars represent ± SD, asterisk indicates the statistical significance of the *t*-test at *p* ≤ 0.05 (N = 3).

The Ni concentration in roots of the NTM ecotype was significantly higher in comparison to the TM ecotype. On the other hand, the Ni concentration in shoots was higher in the TM ecotype (Figure 2).

### 2.2. RNA-seq Analysis of N. goesingensis Root Transcriptomes

**Figure 3:**
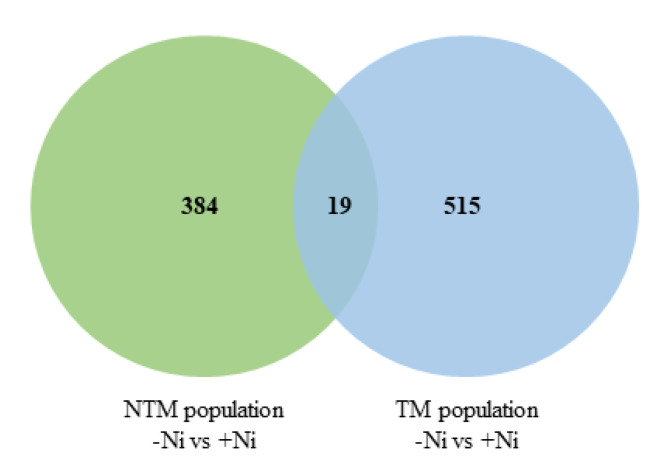
Venn diagram of differentially expressed genes in roots of two *Noccaea goesingensis* ecotypes treated with 150 µM Ni for 21 days. Differential genes expression was evaluated by comparing gene expression profiles of plants grown in the control substrate and in Ni-enriched substrate. Green circle represents the comparison between –Ni vs. +Ni in the Flatz ecotype, blue circle –Ni vs. +Ni in the Redlschlag ecotype.

**Figure 4:**
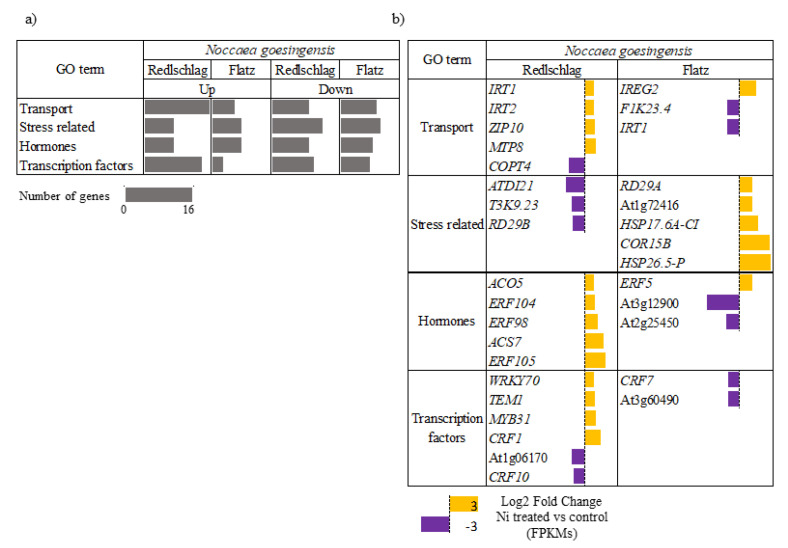
Gene expression analysis of two *Noccaea goesingensis* ecotypes in response to Ni treatment. (**a**) number of differentially expressed genes classified to four functional categories; (**b**) schematic representation of selected genes from each functional category showing their log2 FC of expression after Ni treatment regarding control.

The exposure to Ni altered the expression of 403 and 534 genes in the roots of the NTM and TM ecotypes, respectively. Only 19 genes were commonly regulated in the roots of both ecotypes (Figure 3). Among them, we focused our attention on differentially expressed genes belonging to four categories possibly playing a key role in heavy metal stress responses: transport, stress-related, hormones and transcription factors (Figure 4a). Among all categories, a contrasting response to Ni treatment was observed between both ecotypes.

### 2.3. Up-Regulated Genes 

Among the metal transport category, four genes were upregulated in the TM ecotype, including *IRT1* (IRON-REGULATED TRANSPORTER 1, At419690), *IRT2* (IRON ION TRANSMEMBRANE TRANSPORTER, At4g19680), *ZIP10* (ZINC TRANSPORTER 10 PRECURSOR, At1g31260), *MTP8* (At3g58060) (Figure 4b). In the NTM ecotype, only *IREG2* (IRON-REGULATED PROTEIN 2, At5g03570) (Figure 4b) was up-regulated. Out of the genes classified into the abiotic stress category, five genes were up-regulated in the NTM ecotype: *HSP26.5-P* (26.5 kDa class I small heat shock protein-like, At1g52560), chaperone DnaJ-domain superfamily protein (At1g72416), *HSP17.6A-CI* (17.6 kDa class I heat shock protein, At1g59860), COR15B (COLD REGULATED 15B, At2g42530), *RD29A* (LOW-TEMPERATURE-INDUCED 78, At5g52310). Meanwhile, in the TM ecotype, genes which were identified as abiotic stress-related were down-regulated (Figure 4b). Within the hormone metabolism category, five genes associated with ethylene metabolism were up-regulated in the roots of TM ecotype plants, including ethylene synthesis related genes: *ACS7* (1-AMINOCYCLOPROPANE-1-CARBOXYLATE SYNTHASE, At4g26200), *ACO5* (1-AMINOCYCLOPROPANE-1-CARBOXYLATE OXIDASE, PUTATIVE, At1g77330) and ethylene response related genes: *ERF98* (ETHYLENE-RESPONSIVE FACTOR, PUTATIVE, At3g23230), *ERF104* (ETHYLENE-RESPONSIVE ELEMENT-BINDING FAMILY PROTEIN, At5g61600), *ERF105* (ETHYLENE RESPONSE FACTOR 105, At5g51190) (Figure 4b). In turn, in the NTM ecotype, only *ERF5* (ETHYLENE RESPONSIVE ELEMENT BINDING FACTOR 5, At5g47230) was up-regulated. Within the transcription factors category, 14 genes were up-regulated in the TM ecotype, four of which are described as ethylene-responsive, including *CRF1* (CYTOKININ RESPONSE FACTOR 1, At4g11140) and *WRKY70* (WRKY DNA-BINDING PROTEIN 70, At3g56400) (Figure 4b). In contrast, in the NTM ecotype, four genes were up-regulated in this category; however, none of them was ethylene-responsive (Figure 4b).

### 2.4. Down-Regulated Genes

In the TM ecotype, only one gene was classified into the metal transport category—*COPT4* (COPPER ION TRANSMEMBRANE TRANSPORTER, At2g37925), which was down-regulated (Figure 4b). In turn, in the NTM ecotype, two genes associated with metal transport were down-regulated, i.e., *IRT1* and *F1K23.4* (HEAVY METAL TRANSPORT/DETOXIFICATION SUPERFAMILY PROTEIN, At1g29000) (Figure 4b). Within the abiotic stress category, three genes were down-regulated in the TM ecotype, including *T3K9.23* (HEAT SHOCK PROTEIN BINDING, At2g41000), RD29B (LOW-TEMPERATURE-INDUCED 65, At5g52300) and *ATDI21* (ARABIDOPSIS THALIANA DROUGHT-INDUCED 21, At4g15910) (Figure 4b). None of the genes from this category were down-regulated in the NTM ecotype. Two genes, At3g12900 and At2g25450, associated with response to ethylene, were down-regulated in the NTM ecotype (Figure 4b). In turn, no negative gene regulation in this category was shown in the TM ecotype. Ten genes were down-regulated from the transcription factors category in TM ecotype, two of which belonged to the ethylene-responsive group: At1g06170 and *CRF10* (CYTOKININ RESPONSE FACTOR 10, At1g68550) (Figure 4b). In the NTM ecotype, the expression of eight genes was down-regulated in this category (Figure 4a). Among these genes, only two are associated with the response to ethylene: *CRF7* (CYTOKININ RESPONSE FACTOR 7, At1g22985) and INTEGRASE-TYPE DNA-BINDING SUPERFAMILY PROTEIN (At3g60490) (Figure 4b).

## 3. Discussion

The comparison of the transcriptome of two *N. goesingensis* ecotypes, that differed in Ni accumulation capacity and tolerance, enabled us to provide an insight into mechanisms underlying Ni hyperaccumulation. *Noccaea goesingensis* is considered as a Ni hyperaccumulator and has frequently been studied in terms of Ni tolerance and accumulation [17,18,19,20,22]; however, data concerning the mechanisms involved in Ni hyperaccumulation in this plant species are still scarce. According to our results, plants from the TM population accumulated more Ni in shoots and simultaneously less in roots in comparison to the NTM ecotype, suggesting variation in metal uptake and distribution mechanisms. According to Himmelbauer et al. [22], the root system of *N. goesingensis* exhibits the potential for enhanced Ni uptake from the soil. Furthermore, it is considered that the constitutive overexpression of genes encoding transmembrane transporters in roots, such as members of ZIP and HMA, plays a determinant role in driving the translocation of metals to shoots and is a one of hallmarks distinguishing hyperaccumulators from non-hyperaccumulator plants (reviewed by Rascio et al. [37]). To determine the differences in mechanisms of Ni uptake and translocation between ecotypes, the analysis of the global transcriptome response was performed only in roots.

**Figure 5:**
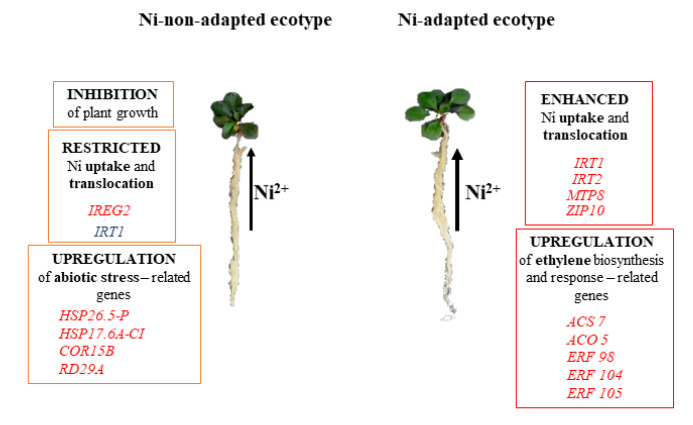
Model of distinct Ni accumulation properties and gene expression patterns in non- and metallicolous ecotypes of *Noccaea goesingensis.* Depicted genes for each ecotype are either upregulated (red) or downregulated (blue). The thickness of the arrow indicates the Ni uptake.

The two *N. goesingensis* populations responded to Ni by modulating the expression of a different gene set, as shown by the small overlap in the number of genes that were regulated by the metal with the same trend of expression (up- or down-regulated), indicating a more qualitative than quantitative variation in plant response (Figure 5).

Among the differentially regulated genes, genes classified into abiotic stress-related category were mainly up-regulated in the roots of the NTM ecotype, whereas in the TM ecotype, genes classified into the same group were down-regulated. This indicates that, although the root biomass of both ecotypes was unaffected by Ni treatment, the NTM ecotype was more prone to metal toxicity, activating mechanisms associated with the stress response. Differences in gene expression were also observed among genes encoding metal transporters, genes associated with ethylene metabolism and genes encoding ethylene-responsive transcription factors. The expression pattern of metal transporter encoding genes suggests that *IRT1*, *IRT2*, *ZIP10*, and *MTP8* may contribute to enhanced Ni accumulation in the TM ecotype. Similar results were described for *N. caerulescens* metallicolous accession [24], where *IRT1*, *IRT2* and *ZIP10* encoding genes were suggested to be associated with the hyperaccumulation of several metals, including Ni. The analogous group of genes, including *IRT1* and *IRT2*, was also up-regulated in *Arabidopsis halleri* Zn hyperaccumulating population [25]. *IRT1* as well as *IRT2* have been shown to possess low substrate specificity, transporting iron but also Zn, Cd and Ni [38,39,40,41]. The low specificity of metal transport applies also to *ZIP10*, which has been shown to transport Fe and Zn [42]. Even slight variations in the transporter sequence may influence the substrate specificity [38], and it remains to be investigated if differentially regulated transporters (including IRT1, IRT2, ZIP10, MTP8) transport Ni more preferably over other metals in the TM *N. goesingensis* ecotype.

The concentration of Ni in shoots of the TM ecotype was higher in comparison to the NTM ecotype, which may indicate changes in the mechanisms associated with Ni distribution in TM ecotype. Up to date, there is a lack of evidence concerning transporters involved in the Ni xylem loading process; however, existing studies suggested the involvement of Zn or/and Fe transport system [43,44]. Among upregulated genes encoding metal transporters, the IRT1 may be considered in the context of Ni distribution, since it was found to translocate other metals, such as Mn in *Hordeum vulgare* [45]. The IRT1 encoding gene was found to be expressed mainly in root epidermal cells in *A. thaliana*, where it participates in metal uptake from the soil [46]. The IRT1 localization in roots of plants from the *Noccaea* genus has not been shown to date, and therefore we cannot exclude a possible role of this transporter in Ni uptake and distribution, which needs further study.

Toxic metal uptake and distribution within plant organisms are under strict control of regulatory pathways mainly controlled by phytohormones, with particular emphasis on ethylene (reviewed by Khan et al., 2017). According to our results, the genes associated with ethylene biosynthesis and response were up-regulated only in the TM ecotype. Additionally, with a view to the above-mentioned upregulation of genes involved in Fe transport, we can hypothesize that ethylene may serve here as a main regulating molecule implicated in the *N. goesingensis* adaptation to Ni excess. We observed the up-regulation of genes involved in both ethylene biosynthesis and ethylene response. However, this consideration is based solely on RNA-seq analysis—the ethylene levels were not determined in the tested ecotypes, and thus this hypothesis requires further verification. There are studies that revealed increased levels of this phytohormone in response to metal presence, including Cd, Cu [47], Ni and Zn [29]. Interestingly, in this latter study, the ethylene-regulated antioxidant metabolism was shown to maintain a higher level of reduced glutathione, alleviating photosynthetic inhibition in metal-exposed mustard plants. Even though recent advances are constantly showing that ethylene plays a pivotal role in heavy metal tolerance (see, for example, reference [48]), more future studies are required to clarify the involvement of ethylene in toxic metal accumulation leading to enhanced tolerance.

Considering that this comparative study was mainly performed at the transcriptomic level, it is worth paying attention to changes observed in transcription factors playing a major role in changes in gene regulatory networks at transcriptional level. The TM population showed a higher number of upregulated transcription factors than NTM. Among them, four (WRKY70, MYB31, TEM1, CRF1) might be key players in the modulation of a signalling cascade of downstream response genes involved in heavy metal hyperaccumulation. WRKY and MYB transcriptional factors have been indicated as master regulators of several stress-related genes [49]. WRKY70, already shown to be upregulated by metal nanoparticles in Arabidopsis [50], is known to be involved in brassinosteroid-regulated plant growth [33], and such hormones can be in turn involved in the heavy metal response [51]. MYB31 is thought to act as a repressor of lignin biosynthesis [52] and was found to be responsive to auxin [53]; nonetheless, to our knowledge, it has never been linked to plant metal response. Together with the abovementioned induction of TEM1, involved in the ethylene-mediated response, the up-regulation of these transcription factors could suggest the involvement of a different hormonal scenario in the TM population with respect to the NTM one. In addition to such transcription factors, our results could suggest CRF1 as another interesting candidate, worthy of further investigated in the context of Ni hyperaccumulation, as it is known to be involved in heavy metal response in eukaryotic organisms, mediating copper and cadmium resistance in the yeast *Yarrowia lipolytica* [54].

In conclusion, the performed studies revealed clear differences in the response to Ni in TM and NTM *N. goesingensis* ecotypes at the level of accumulation capacity and in the whole transcriptome response in roots. The study allowed us to indicate increased expression of major genes encoding metal transporters, *IRT1* and *IRT2*, and involved in ethylene biosynthesis and signalling, *ACS7*, *ACO5*, *ERF104*, and *ERF105*, probably associated with Ni hyperaccumulation in contrasting populations of *N. goesingensis*.

## 4. Materials and Methods

### 4.1. Plant Cultivation

Seeds of *Noccaea goesingensis* were collected from two populations: dolomite (NTM) Flatz (47.746628° N, 16.010337° E) and serpentinite (TM) Redlschlag (47.436694° N, 16.278729° E) in Austria. Seeds were collected from several plants from each population and surface sterilized in 8% sodium hypochlorite for 5 min, followed by 75% ethanol for 1 min and 96% ethanol for 3 min, and washed 5 times with sterile deionized water. Subsequently, seeds were sown into sterile substrate composed of a mixture of sand and perlite (1:2, *v:v*), stratified for 2 days at 4 °C in darkness and germinated for 12 days in a plant growth chamber (Biogenet FITO700, Poland) with a 16 h photoperiod, under 190 µmol · m^−2^ · s^−1^ of light intensity, 24/19 °C day/night temperature and 70% humidity. After 12 days, plants were transferred into 50 mL pots (one plant per pot, n = 30 for each experimental group: NTM, NTM Ni+, TM, TM Ni+) filled with the same substrate and cultivated under the same conditions. Plants were irrigated two times per week with 6 mL of sterile deionized water and one time per week with 6 mL of sterile Hoagland solution (2 mM MgSO_4_ · 7 H_2_O, 0.8 mM Ca(NO_3_)_2_, 2.5 mM KNO_3_, 0.1 mM K_2_HPO_4_, 20 μM FeEDDHA, 10 μM H_3_BO_3_, 2 μM MnCl_2_, 1 μM ZnSO_4_, 0.5 μM CuSO_4_, 0.2 μM Na_2_MoO_4_). After 14 days of growth, plants were irrigated with sterile Hoagland solution supplemented with 150 µM of NiSO_4_ · 5 H_2_O once per week except for the control group (-Ni), which was irrigated simultaneously with the Hoagland solution without Ni. The treatment with Ni lasted three weeks; after this time, plants were harvested and weighed (dose in total ca. 2700 µM). Fifteen plants from each experimental group were frozen in liquid nitrogen for RNA extraction, and the remaining 15 plants were dried at 80 °C for 24 h for dry biomass determination and metal analysis.

### 4.2. Plant Nickel Concentration

Approximately 50 mg of root and shoot samples (N = 3) were weighted to analytical accuracy, transferred into teflon autoclave (Speedwave^®^ Entry, Berghof, Germany) and pre-digested in 5.00 mL of 65% HNO_3_ (Argenta, Poland) at room temperature for 1 h. The digestion was carried out for 30 min after the addition of 30% hydrogen peroxide (Sigma Aldrich, Saint Louis, MO, USA). Microwave digestion was carried out for 35 min (temp profile: step 1—ramp 5 °C/min, time—5 min, temp: 145 °C; step 2—ramp 3 °C/min, time—10 min, temp: 190 °C; step 3—ramp 10 °C/min, time—1 min, temp: 75 °C). Subsequently, the solution was cooled to room temperature and quantitatively transferred into a 25 mL volumetric flask and made up with deionized water. The blank samples were processed simultaneously according to the same analytical procedure. Nickel was measured by using atomic absorption spectrometry (graphite furnace [GF-AAS], equipped with an auto-sampler [Thermo Scientific, iC3000, US]). The external standard calibration method was applied using AAS standard solutions (Sigma Aldrich, USA). All chemicals were trace metal grade.

### 4.3. RNA Sequencing and Functional Data Mining

Total RNA Mini Kit (Bio-Rad, Hercules, CA, USA) was used for total RNA extraction from roots (from 5 plants per sample, N = 3). RNA digestion was performed with DNase (DNA free kit, Ambion Bioscience, Austin, TX USA) and RNA purity and quantity were analyzed by Biospec-Nano (SHIMADZU, Japan). The integrity of RNA was assessed with the Agilent 2100 Bioanalyzer (Agilent, Santa Clara, CA, USA) and RNA 6000 Nano Kit (Agilent, Santa Clara, CA, USA). Ion Total RNA-seq Kit v2 (ThermoFisher Scientific, Waltham, MA, USA) was utilized for whole transcriptome libraries preparation. Poly(A) RNA selection was performed with 1000 ng of total RNA with the use of Dynabeads mRNA DIRECT Micro Kit (ThermoFisher Scientific, Waltham, MA, USA) following the manufacturer’s protocol. Next, the poly(A) RNA was fragmented with RNase III, purified and checked for the quality with Agilent 2100 Bioanalyzer (Agilent Technologies, Santa Clara, CA, USA) and the RNA 6000 Pico Kit (Agilent Technologies, Santa Clara, CA, USA). In the next step, the RNA was hybridized and ligated. Reverse transcription was carried out using the Ion Total RNA-seq Kit v2 and the cDNA was purified, amplified and barcoded with the Ion Xpress RNA-Seq Barcode 1–16 Kit (ThermoFisher Scientific, Waltham, MA, USA) and purified. The High Sensitivity DNA Kit with the use of the 2100 Bioanalyzer (Agilent Technologies, Santa Clara, CA, USA) was utilized for yield and size distribution of amplified DNA determination. All of the prepared libraries were diluted to equimolar concentrations (100 pM) and merged into sets of 6 samples. Template-positive ion (PI) ion sphere particles (ISPs) with 200 base-pair average insert libraries for sequencing were prepared and enriched using Ion PI Hi-Q OT2 200 Kit (ThermoFisher Scientific, Waltham, MA, USA) and Ion OneTouch 2 System (ThermoFisher Scientific, USA) according to the manufacturer’s protocol. Sequencing was conducted with the use of Ion PI Hi-Q Sequencing 200 Kit (ThermoFisher Scientific, Waltham, MA, USA) and Ion PI Chip Kit v3 (ThermoFisher Scientific, Waltham, MA, USA) on the Ion Proton System (ThermoFisher Scientific, Waltham, MA, USA) according to the standard protocol provided by the manufacturer. The raw data were processed by trimming low quality bases and the adaptor sequence was removed to obtain high-quality clean reads using cutadapt version 1.8.1. The pre-processed high-quality reads were mapped to *A. thaliana* genome with HISAT2 version 2.1.0 [55] using the default parameters. The alignment statistics for the same are given in Table S1. The resulting output of HISAT2 was employed to determine the differentially expressed genes using Cuffdiff tool in Cufflinks version 2.2.1 pipeline with default parameters. We considered up- and down-regulated genes with a *p*-value < 0.05 and an absolute value of log2FC > 0.05, and they were used for downstream functional analysis. The Differentially Expressed Genes (DEGs) selected were annotated using the *A. thaliana* genome. The differentially expressed genes were called upon by comparing Ni with non-Ni-treated libraries separately for the TM and NTM ecotypes. Using the p.adjust function of R, all the statistical tests were corrected for multiple comparisons using the Benjamini–Hochberg false discovery rate [56]. Differences among the selected studies were adjusted using the sample normalization. To remove systematic variation between different species, the normalization procedure served as a crucial pre-processing step to adjust for the different sample sequencing depths and other confounding technical effects. We used the geometric normalization method where Fragments Per Kilobase Million (FPKMs) and fragment counts are scaled via the median of the geometric means of fragment counts across all libraries. All the differentially regulated genes IDs of *N. goesingensis* were mapped to closely related *A. thaliana* genome alignment (an overall sequence identity of ca. 86%, based on ITS1 and ITS2 [57]) and the corresponding best hit TAIR ID (hypothetical ortholog) was determined using the annotation file downloaded from Phytozome. MapMan [58] was used with the *A. thaliana* mapping file to map and visualize the metabolic overview, hormone regulation, transcription factors, and transport-related categories. Approximately 65% of *N. goesingensis* reads were mapped to *A. thaliana* genome (TAIR). The comparison was performed within each genotype (TM and NTM) between nickel treatment and the control condition. Up- and down-regulated genes were, respectively, more and less expressed in response to nickel presence.

### 4.4. Data Availability

The RNA-seq data reported in this article were deposited in NCBI’s Gene Expression Omnibus (GEO) and are accessible through GEO Series accession number GSE155054.

### 4.5. Statistical Analysis

Statistical analysis was performed using Statistica ver. 13.0 (StatSoft) for all the obtained data. Normality of all data was evaluated by the Shapiro–Wilk test. Variance homogeneity was tested by Levene’s test. For pairwise comparisons, the *t*-Student test was used.

## Supplementary Materials

The following are available online at https://www.mdpi.com/2223-7747/9/8/951/s1, Table S1: The number of reads for each ecotype/treatment combination and the number of reads successfully mapped to the reference genome vs. the number of reads not mapped.

## References

[1] Baker A.J.M., Ernst W.H.O., van der Ent A., Malaisse F., Ginocchio R. (2012). Metallophytes: The unique biological resource, its ecology and conservational status in Europe, central Africa and Latin America. Ecol. Ind. Pollut..

[2] Pauwels M., Vekemans X., Godé C., Frérot H., Castric V., Saumitou-Laprade P. (2012). Nuclear and chloroplast DNA phylogeography reveals vicariance among European populations of the model species for the study of metal tolerance, Arabidopsis halleri (Brassicaceae). New Phytol..

[3] Ernst W.H.O. (2006). Evolution of metal tolerance in higher plants. For. Snow Landsc. Res..

[4] Wójcik M., Gonnelli C., Selvi F., Dresler S., Rostański A., Vangronsveld J. (2017). Metallophytes of Serpentine and Calamine Soils—Their Unique Ecophysiology and Potential for Phytoremediation. Adv. Bot. Res..

[5] Lambinon J., Auquier P. (1964). La flore et la végétation des terrains calaminaires de la Wallonie septentrionale et de la Rhénanie aixoise. Types chorologiques et groupes écologiques. Nat. Mosana.

[6] Willems G., Dräger D.B., Courbot M., Godé C., Verbruggen N., Saumitou-Laprade P. (2007). The Genetic Basis of Zinc Tolerance in the Metallophyte Arabidopsis halleri ssp. halleri (Brassicaceae): An Analysis of Quantitative Trait Loci. Genetics.

[7] Bothe H., Sherameti I., Varma A. (2011). Plants in Heavy Metal Soils. Soil biology. Detoxification of Heavy Metals.

[8] Bothe H., Słomka A. (2017). Divergent biology of facultative heavy metal plants. J. Plant Physiol..

[9] Krämer U. (2010). Metal Hyperaccumulation in Plants. Annu. Rev. Plant Biol..

[10] Reeves R.D., Baker A.J.M., Jaffré T., Erskine P.D., Echevarria G., van der Ent A. (2018). A global database for plants that hyperaccumulate metal and metalloid trace elements. New Phytol..

[11] Reeves R.D., Brooks R.R. (1983). European species of Thlaspi L. (Cruciferae) as indicators of nickel and zinc. J. Geochem. Explor..

[12] Persans M.W., Yan X., Patnoe J.M.M.L., Krämer U., Salt D.E. (1999). Molecular dissection of the role of histidine in nickel hyperaccumulation in Thlaspi goesingense (Halacsy). Plant Physiol..

[13] Rosenkranz T., Hipfinger C., Ridard C., Puschenreiter M. (2019). A nickel phytomining field trial using Odontarrhena chalcidica and Noccaea goesingensis on an Austrian serpentine soil. J. Environ. Manag..

[14] Krämer U., Smith R.D., Wenzel W.W., Raskin I., Salt D.E. (1997). The role of metal transport and tolerance in nickel hyperaccumulation by Thlaspi goesingense Halacsy. Plant Physiol..

[15] Reeves R.D., Baker A.J.M. (1984). Studies on metal uptake by plants from serpentine and non-serpentine populations of Thlaspi goesingense halácsy (Crycuferae). New Phytol..

[16] Kidd P.S., Bani A., Benizri E., Gonnelli C., Hazotte C., Kisser J., Konstantinou M., Kuppens T., Kyrkas D., Laubie B. (2018). Developing Sustainable Agromining Systems in Agricultural Ultramafic Soils for Nickel Recovery. Front. Environ. Sci..

[17] Krämer U., Pickering I.J., Prince R.C., Raskin I., Salt D.E. (2000). Subcellular Localization and Speciation of Nickel in Hyperaccumulator and Non-Accumulator Thlaspi Species. Plant Physiol..

[18] Küpper H., Lombi E., Zhao F., Wieshammer G., McGrath S.P. (2001). Cellular compartmentation of nickel in the hyperaccumulators Alyssum lesbiacum, Alyssum bertolonii and Thlaspi goesingense. J. Exp. Bot..

[19] Persans M.W., Nieman K., Salt D.E. (2001). Functional activity and role of cation-efflux family members in Ni hyperaccumulation in Thlaspi goesingense. Proc. Natl. Acad. Sci. USA.

[20] Freeman J.L., Persans M.W., Nieman K., Albrecht C., Peer W., Pickering I.J., Salt D.E. (2004). Increased Glutathione Biosynthesis Plays a Role in Nickel Tolerance in Thlaspi Nickel Hyperaccumulators. Plant Cell.

[21] Freeman J.L., Salt D.E. (2007). The metal tolerance profile of Thlaspi goesingense is mimicked in Arabidopsis thaliana heterologously expressing serine acetyl-transferase. BMC Plant Biol..

[22] Himmelbauer M.L., Puschenreiter M., Schnepf A., Loiskandl W., Wenzel W.W. (2005). Root morphology of Thlaspi goesingense Hálácsy grown on a serpentine soil. J. Plant Nutr. Soil Sci..

[23] Plessl M., Rigola D., Hassinen V.H., Tervahauta A., Kärenlampi S., Schat H., Aarts M.G.M., Ernst D. (2010). Comparison of two ecotypes of the metal hyperaccumulator Thlaspi caerulescens (J. & C. PRESL) at the transcriptional level. Protoplasma.

[24] Halimaa P., Lin Y.F., Ahonen V.H., Blande D., Clemens S., Gyenesei A., Häikiö E., Kärenlampi S.O., Laiho A., Aarts M.G.M. (2014). Gene expression differences between noccaea caerulescens ecotypes help to identify candidate genes for metal phytoremediation. Environ. Sci. Technol..

[25] Schvartzman M.S., Corso M., Fataftah N., Scheepers M., Nouet C., Bosman B., Carnol M., Motte P., Verbruggen N., Hanikenne M. (2018). Adaptation to high zinc depends on distinct mechanisms in metallicolous populations of Arabidopsis halleri. New Phytol..

[26] Chmielowska-Bąk J., Lefèvre I., Lutts S., Deckert J. (2013). Short term signaling responses in roots of young soybean seedlings exposed to cadmium stress. J. Plant Physiol..

[27] Maksymiec W. (2011). Effects of jasmonate and some other signalling factors on bean and onion growth during the initial phase of cadmium action. Biol. Plant..

[28] Masood A., Iqbal N., Khan N.A. (2012). Role of ethylene in alleviation of cadmium-induced photosynthetic capacity inhibition by sulphur in mustard. Plant. Cell Environ..

[29] Khan M.I.R., Khan N.A. (2014). Ethylene reverses photosynthetic inhibition by nickel and zinc in mustard through changes in PS II activity, photosynthetic nitrogen use efficiency, and antioxidant metabolism. Protoplasma.

[30] Khan N.A., Khan M.I.R., Ferrante A., Poor P. (2017). Editorial: Ethylene: A Key Regulatory Molecule in Plants. Front. Plant Sci..

[31] Cao S., Chen Z., Liu G., Jiang L., Yuan H., Ren G., Bian X., Jian H., Ma X. (2009). The Arabidopsis Ethylene-Insensitive 2 gene is required for lead resistance. Plant Physiol. Biochem..

[32] Monteiro C.C., Carvalho R.F., Gratão P.L., Carvalho G., Tezotto T., Medici L.O., Peres L.E.P., Azevedo R.A. (2011). Biochemical responses of the ethylene-insensitive Never ripe tomato mutant subjected to cadmium and sodium stresses. Environ. Exp. Bot..

[33] Chen J., Nolan T., Ye H., Zhang M., Tong H., Xin P., Chu J., Chu C., Li Z., Yin Y. (2017). Arabidopsis WRKY46, WRKY54 and WRKY70 Transcription Factors Are Involved in Brassinosteroid-Regulated Plant Growth and Drought Response. Plant Cell.

[34] Lingam S., Mohrbacher J., Brumbarova T., Potuschak T., Fink-Straube C., Blondet E., Genschik P., Bauer P. (2011). Interaction between the bHLH transcription factor FIT and ETHYLENE INSENSITIVE3/ETHYLENE INSENSITIVE3-LIKE1 reveals molecular linkage between the regulation of iron acquisition and ethylene signaling in Arabidopsis. Plant Cell.

[35] Halimaa P., Blande D., Baltzi E., Aarts M.G.M., Granlund L., Keinänen M., Kärenlampi S.O., Kozhevnikova A.D., Peräniemi S., Schat H. (2019). Transcriptional effects of cadmium on iron homeostasis differ in calamine accessions of Noccaea caerulescens. Plant J..

[36] Chen Q., Wu K., Tang Z., Guo Q., Guo X., Wang H. (2017). Exogenous ethylene enhanced the cadmium resistance and changed the alkaloid biosynthesis in Catharanthus roseus seedlings. Acta Physiol. Plant..

[37] Rascio N., Navari-Izzo F. (2011). Heavy metal hyperaccumulating plants: How and why do they do it? And what makes them so interesting?. Plant Sci..

[38] Rogers E.E., Eide D.J., Guerinot M.L. (2000). Altered selectivity in an Arabidopsis metal transporter. Proc. Natl. Acad. Sci. USA.

[39] Nishida S., Tsuzuki C., Kato A., Aisu A., Yoshida J., Mizuno T. (2011). AtIRT1, the primary iron uptake transporter in the root, mediates excess nickel accumulation in Arabidopsis thaliana. Plant Cell Physiol..

[40] Korshunova Y.O., Eide D., Gregg Clark W. (1999). The IRT1 protein from Arabidopsis thaliana is a metal transporter with a broad substrate range. Plant Mol. Biol..

[41] Vert G., Briat J.-F., Curie C. (2001). Arabidopsis IRT2 gene encodes a root-periphery iron transporter. Plant J..

[42] Talke I.N., Hanikenne M., Krämer U. (2006). Zinc-Dependent Global Transcriptional Control, Transcriptional Deregulation, and Higher Gene Copy Number for Genes in Metal Homeostasis of the Hyperaccumulator Arabidopsis halleri. Plant Physiol..

[43] Assuncao A.G.L., Martins P.D.C., De Folter S., Vooijs R., Schat H., Aarts M.G.M. (2001). Elevated expression of metal transporter genes in three accessions of the metal hyperaccumulator Thlaspi caerulescens. Plant Cell Environ..

[44] Ghasemi R., Ghaderian S.M., Krämer U. (2009). Interference of nickel with copper and iron homeostasis contributes to metal toxicity symptoms in the nickel hyperaccumulator plant Alyssum inflatum. New Phytol..

[45] Long L., Persson D.P., Duan F., Jørgensen K., Yuan L., Schjoerring J.K., Pedas P.R. (2018). The iron-regulated transporter 1 plays an essential role in uptake, translocation and grain-loading of manganese, but not iron, in barley. New Phytol..

[46] Dubeaux G., Neveu J., Zelazny E., Vert G. (2018). Metal Sensing by the IRT1 Transporter-Receptor Orchestrates Its Own Degradation and Plant Metal Nutrition. Mol. Cell.

[47] Arteca R.N., Arteca J.M. (2007). Heavy-metal-induced ethylene production in Arabidopsis thaliana. J. Plant Physiol..

[48] Thao N.P., Khan M.I.R., Anh Thu N.B., Thi Hoang X.L., Asgher M., Khan N.A., Tran L.S.P. (2015). Role of ethylene and its cross talk with other signaling molecules in plant responses to heavy metal stress. Plant Physiol..

[49] Baillo E.H., Kimotho R.N., Zhang Z., Xu P. (2019). Transcription Factors Associated with Abiotic and Biotic Stress Tolerance and Their Potential for Crops Improvement. Genes.

[50] Landa P., Vankova R., Andrlova J., Hodek J., Marsik P., Storchova H., White J.C., Vanek T. (2012). Nanoparticle-specific changes in Arabidopsis thaliana gene expression after exposure to ZnO, TiO2, and fullerene soot. J. Hazard. Mater..

[51] Rajewska I., Talarek M., Bajguz A. (2016). Brassinosteroids and Response of Plants to Heavy Metals Action. Front. Plant Sci..

[52] Fornalé S., Shi X., Chai C., Encina A., Irar S., Capellades M., Fuguet E., Torres J.-L., Rovira P., Puigdomènech P. (2010). ZmMYB31 directly represses maize lignin genes and redirects the phenylpropanoid metabolic flux. Plant J..

[53] Goda H., Sawa S., Asami T., Fujioka S., Shimada Y., Yoshida S. (2004). Comprehensive Comparison of Auxin-Regulated and Brassinosteroid-Regulated Genes in Arabidopsis. Plant Physiol..

[54] García S., Prado M., Dégano R., Domínguez A. (2002). A Copper-responsive Transcription Factor, CRF1, Mediates Copper and Cadmium Resistance in Yarrowia lipolytica. J. Biol. Chem..

[55] Kim D., Langmead B., Salzberg S.L. (2015). HISAT: A fast spliced aligner with low memory requirements. Nat. Methods.

[56] Benjamini Y., Hochberg Y. (1995). Controlling the False Discovery Rate: A Practical and Powerful Approach to Multiple Testing. J. R. Stat. Soc. Ser. B.

[57] Peer W.A., Mamoudian M., Lahner B., Reeves R.D., Murphy A.S., Salt D.E. (2003). Identifying model metal hyperaccumulating plants: Germplasm analysis of 20 Brassicaceae accessions from a wide geographical area. New Phytol..

[58] Thimm O., Bläsing O., Gibon Y., Nagel A., Meyer S., Krüger P., Selbig J., Müller L.A., Rhee S.Y., Stitt M. (2004). Mapman: A user-driven tool to display genomics data sets onto diagrams of metabolic pathways and other biological processes. Plant J..

